# The microbiome of deep-sea fish reveals new microbial species and a sparsity of antibiotic resistance genes

**DOI:** 10.1080/19490976.2021.1921924

**Published:** 2021-05-10

**Authors:** Fergus W. J. Collins, Calum J. Walsh, Beatriz Gomez-Sala, Elena Guijarro-García, David Stokes, Klara B. Jakobsdóttir, Kristján Kristjánsson, Finlay Burns, Paul D. Cotter, Mary C. Rea, Colin Hill, R. Paul Ross

**Affiliations:** a Teagasc Food Research Centre, Fermoy, Ireland; b APC Microbiome Ireland, University College Cork, Ireland; c Department of Microbiology, University College Cork, Cork, Ireland; d Spanish Oceanography Institute, Spain; e Marine Institute, Oranmore, Ireland; f Marine and Freshwater Research Institute, Reykjavik, Iceland; g Marine Scotland-Science, Aberdeen, UK

**Keywords:** Deep-sea, microbiome, antibiotic resistance

## Abstract

Adaptation to life in the deep-sea can be dramatic, with fish displaying behaviors and appearances unlike those seen in any other aquatic habitat. However, the extent of which adaptations may have developed at a microbial scale is not as clear. Shotgun metagenomic sequencing of the intestinal microbiome of 32 species of deep-sea fish from across the Atlantic Ocean revealed that many of the associated microbes differ extensively from those previously identified in reference databases. 111 individual metagenome-assembled genomes (MAGs) were constructed representing individual microbial species from the microbiomes of these fish, many of which are potentially novel bacterial taxa and provide a window into the microbial diversity in this underexplored environment. These MAGs also demonstrate how these microbes have adapted to deep-sea life by encoding a greater capacity for several cellular processes such as protein folding and DNA replication that can be inhibited by high pressure. Another intriguing feature was the almost complete lack of genes responsible for acquired resistance to known antibiotics in many of the samples. This highlights that deep-sea fish microbiomes may represent one of few animal-associated microbiomes with little influence from human activity. The ability of the microbes in these samples to bioluminesce is lower than expected given predictions that this trait has an important role in their life cycle at these depths. The study highlights the uniqueness, complexity and adaptation of microbial communities living in one of the largest and harshest environments on Earth.

## Introduction

The deep-sea is an environment characterized by lack of light, low temperatures, high pressure and low nutrient levels, and despite being one of the largest habitats on earth, the deep-sea remains out of reach for many researchers^1^. The deep sea has concealed novel life forms at both a macroscopic and microscopic scale. It has been estimated that the deep sea harbors one of the largest pools of microbes in aquatic systems, accounting for almost 75% of the oceanic prokaryotic biomass,^2^ but its microbial composition is vastly underexplored compared to surface water and terrestrial environments.^3^

Metagenomic sequencing approaches are particularly suitable for the deep sea since they can characterize the entire microbial population within a sample without the need for microbiological culturing. Marine metagenomic studies have focused on the characterization of microbial communities at different depths in the water column, both as particle-attached and free-living communities.^4–6^ The intestines of fish represent one of the densest nutrient sources in the deep and so the fish gut microbiome is likely to be one of the most concentrated bacterial communities in this vast and highly dilute environment. Several studies have investigated the composition of the intestinal microbiota in marine fish, but these have primarily focused on fish of commercial interest and at relatively shallow depths.^7,8^ The composition of the microbiome of deep-sea fish is likely to be unique given the environmental challenges faced by both host and microbes, particularly in terms of temperature, pressure and nutrient availability. The microbiota of fish undoubtedly plays a vital role in their health and development, symbiotically contributing through microbial digestion releasing metabolites that can be utilized by the host.^9^

**Figure 1.**
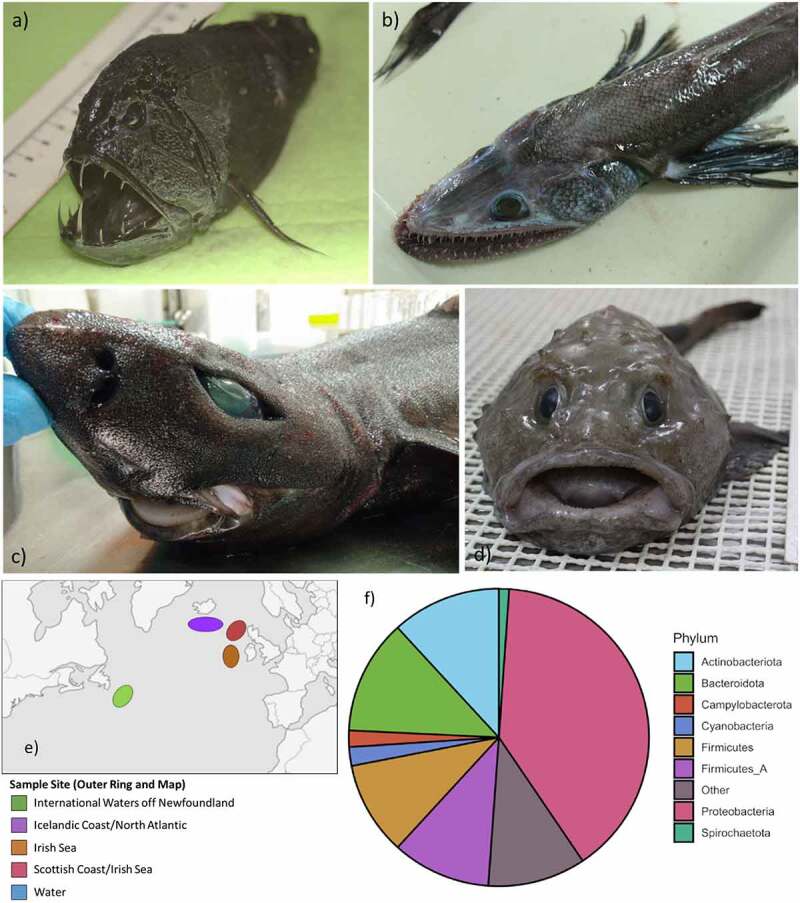
**Summary of sample of collection and dataset**. a-d) Example of some of the deep-sea species subjected to shotgun metagenomic sequencing of their intestinal microbiome – *Anoplogaster cornuta, Bathysaurus ferox, Centroscymnus coelolepis*, and *Cottunculus thomsonii* respectively. e) Sampling locations. f) Phylum-level classification of metagenomic data excluding water sample

Here we used shotgun metagenomic sequencing to characterize the gut microbiomes of 47 fish, representing 32 species, caught at a depth of approximately 1000 m with a corresponding pressure of ~10 MPa (**Supplemental Table 1**). Fish were caught by research vessels in surveys from the offshore waters of Ireland, Scotland and Iceland and in international waters off the Grand Banks of Newfoundland, with a water sample also taken off the Irish coast (Figure 1). Due to difficulties in sampling, it was not possible to obtain water samples from the other sites. Shotgun metagenomic sequencing was subsequently performed on the gut contents, and we were able to reconstruct 111 metagenome-assembled genomes (MAGs). This allowed us to analyze the diversity and functional properties of individual microbes isolated from this environment. A comparison between deep-sea bacteria and their most closely related reference genomes allowed us to identify potential adaptations required for life at these depths. We also examined the distribution of bioluminescence-associated genes in deep-sea microbes. A search for antibiotic resistance genes provided an opportunity to assess their distribution in an environment that has not been exposed to commercial antibiotics.

## Results and discussion

### Compositional and MAG analysis

We used the taxonomic classification tool Kraken2 to determine the microbial composition of the intestinal microbiome from shotgun metagenomic data.^10^ While efforts were made to deplete potential reads from the host DNA itself, these, along with reads associated with host diet, are still likely to account for a portion of the large number of unclassified reads from these samples – reported by Kraken2 as 82.7 ± 1.5%. To minimize the potential for misclassification of Eukaryotic sequences, we utilized a Kraken2 database based on the Genome Taxonomy Database (GTDB) that, in addition to employing a revised and restructured taxonomy system, contains prokaryotic genome sequences only. Of the reads that could be classified, Proteobacteria, Actinobacteria and Bacteriodota are the dominant phyla in these fish, comprising a mean relative abundance of 63.03 ± 3.86% with Firmicutes and Firmicutes_A, a newly proposed phylum composed of taxa formerly classified as Firmicutes, present at lower relative abundances (10.35 ± 0.5% and 10.86 ± 0.46%, respectively) (Figure 1f). Whilst Proteobacteria and Firmicutes can be typically prominent in the microbiome of marine fish, the sheer quantity of unknown reads highlights the uniqueness of these microbiomes compared to those of other marine fish previously studied.^9^ Although the composition of these microbiomes may be difficult to fully elucidate due to the uniqueness of their constituents, by looking at the genes these microbes encode it is possible to garner insights into their functional potential and adaptations to the deep.

The assembly of near-complete bacterial genomes from this metagenomic data allows us obtain information on some of these individual microbes and affords an opportunity to identify some of the adaptations they have evolved to survive in this environment. MAGs are created by grouping contigs from the metagenomic data that are likely to be from the same strain of microbe into “bins” that can then be used to assemble a representative genome.^11,12^ Whilst MAGs lack the purity and completion of individually sequenced bacterial genomes, they do provide information about strains that previously may not have been possible to isolate or sequence.^13^

**Figure 2.**
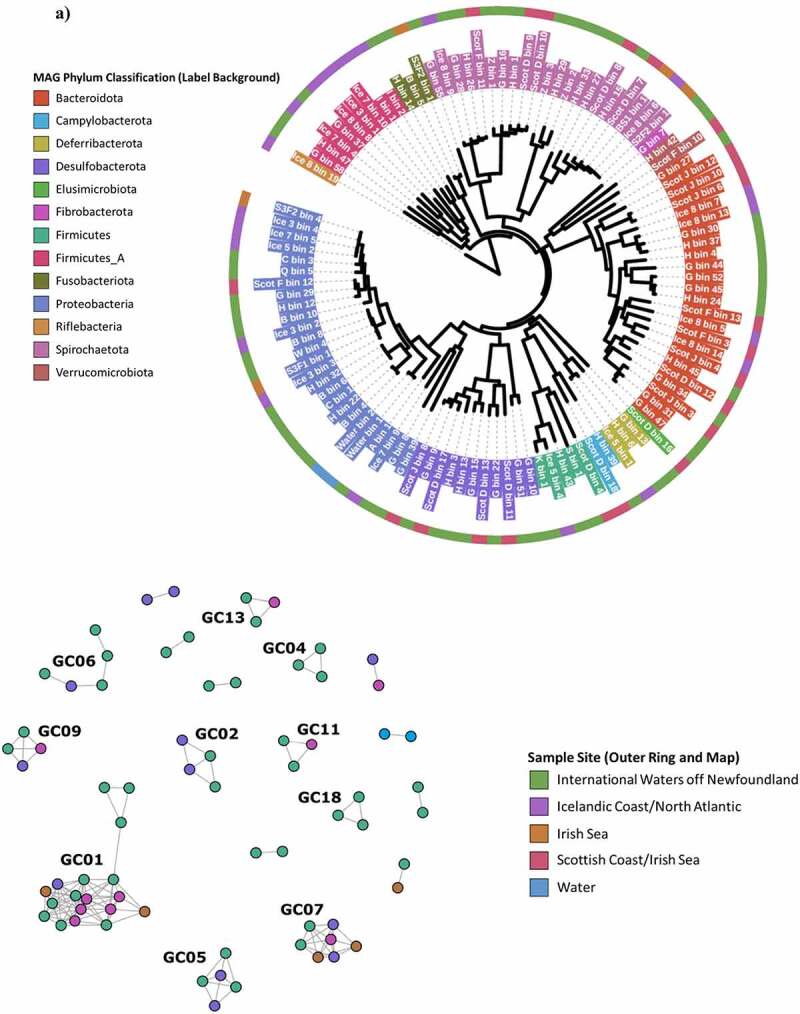
**Distribution of diversity of metagenome-assembled genomes**. a) Phylogenetic tree of bacterial MAGs built by GTDB-tk. Leaf label backgrounds represent MAG phylum also assigned by GTDB-tk. b) Network analysis demonstrating protein-content similarity of metagenome-assembled genomes. Each filled dot (node) represents a metagenome-assembled genome. Nodes are colored by sampling location and linked if they possess Percentage of Conserved Proteins (POCP) > 50% – a genus-level boundary proposed by Qin *et al*.^14^ Intra-cluster physical positioning is representative of POCP (closer means more similar). Inter-cluster positioning is random. Genus clusters are classified by connected subgraph components. Only clusters with 3 or more members are labeled and only those with 2 or more members are shown

Assembly and binning of reads from the deep-sea metagenomic data was performed independently for each of the 47 intestinal microbiome samples, as well as from a water sample taken at >1000 m deep from the Irish coast, resulting in the creation of 111 high-quality MAGs (Figure 2). Of these 111 MAGs, 52 could be classified to genus-level and only 2 assigned a species (**Supplementary Table 2**). This highlights the unexplored biological diversity represented by these MAGs that potentially constitute numerous novel taxa. Grouping these MAGs into Genus Clusters based on percentage of conserved proteins (POCP) shows that taxonomically similar MAGs can be isolated from different fish species caught in separate expeditions in distant parts of the Atlantic Ocean and further highlights the successful adaption of these microbes to life in the deep-sea (Figure 2b**, Supplemental Table 3**).

**Figure 3.**
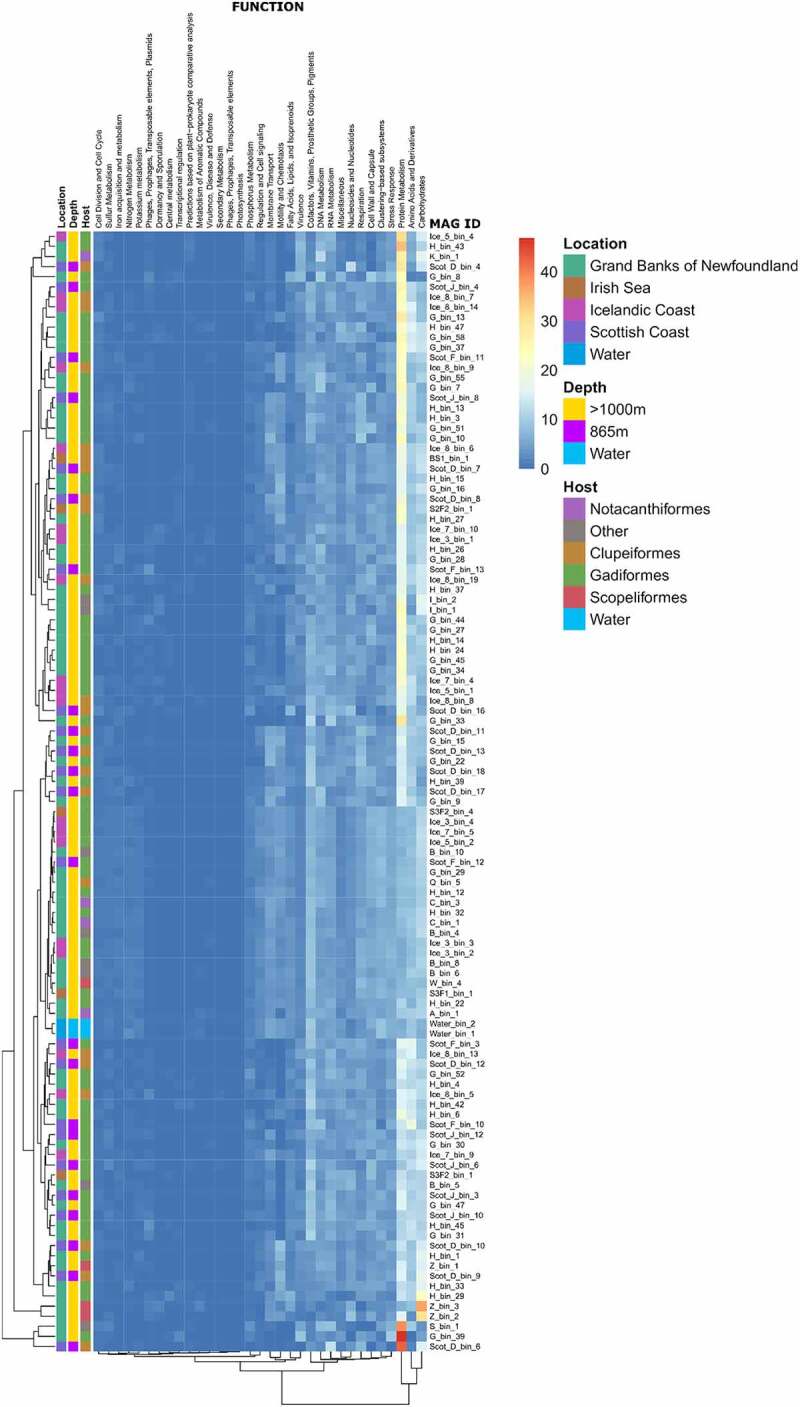
Visualization of functional diversity amongst MAGs assembled using SUPER-FOCUS level 1 results

The abundance of these newly identified MAGs was then assessed across the 47 samples analyzed in this study. Interestingly, all the novel MAGs combined are present at an abundance of <5% in two thirds of the samples analyzed (**Supplemental Table 4**), suggesting they are dominant members of the microbiome in some of these fishes but form part of the rare biosphere in others. While many of the MAGs cannot be classified to a genus or species, we can obtain information on the functional capacity of these novel microbes. We used SUPER-FOCUS to carry out a functional trait comparison across all 111 MAGs. The results indicate that functionally dissimilar microbes co-exist in these similar environments (Figure 3). This differentiation of traits may allow these microbes to co-exist in the intestine of these fishes, with each occupying its own niche, or functioning synergistically within communities.

In order to identify possible adaptations of these deep-sea microbes, SUPER-FOCUS was also used to compare the functional properties of those MAGs with closely related reference genomes in the RefSeq and MarDB databases.^15^ From the 111 MAGs constructed, only 39 had at least 75% average nucleotide identity (ANI) to a reference genome (Table 1). Visualization of the functional diversity on a PCoA (principal coordinate analysis) plot shows how these MAGs have diverged from their closest relative (**Supplemental Fig. 1**). Interestingly, many of the MAGs identified that do not have a related reference genome are very distant from the other MAGs and reference genomes, again highlighting the apparent diversity in the lifestyle of these MAGs.

**Table 1.** MAGs and reference genomes with an average nucleotide identity >75% used for SUPER-FOCUS comparison

MAG ID (Size Mbp)	Closest Reference Genome (Size Mbp)	ANI (%)
A_bin_1 (4.11)	*Endozoicomonas elysicola* DSM 22380 (5.61)	82.5
B_bin_10 (2.98)	*Enterovibrio calviensis* 1 F 230 (5.46)	78.8
B_bin_4 (3.09)	*Psychromonas arctica* DSM 14288 (4.75)	79.4
B_bin_5 (2.68)	*Psychrilyobacter atlanticus* DSM 19335 (3.54)	76.8
B_bin_6 (3.23)	*Moritella dasanensis* ArB 0140 (4.89)	85.3
B_bin_8 (3.96)	*Photobacterium phosphoreum* (4.55)	86.5
BS1_bin_1 (2.47)	*Brachyspira hyodysenteriae* (3.02)	76.2
C_bin_1 (4.39)	*Moritella dasanensis* ArB 0140 (4.89)	85.4
C_bin_3 (2.83)	*Enterovibrio calviensis* 1 F 211 (5.60)	77.7
G_bin_29 (3.88)	*Enterovibrio calviensis* DSM 14347 (5.50)	77.3
H_bin_12 (3.29)	*Grimontia celer* (5.61)	77.1
H_bin_14 (3.44)	*Fusobacterium mortiferum* ATCC 9817 (2.72)	76.6
H_bin_15 (3.44)	*Brachyspira intermedia* PWS A (3.3)	75.7
H_bin_22 (1.97)	*Psychromonas arctica* DSM 14288 (4.75)	80.1
H_bin_27 (2.04)	*Brachyspira pilosicoli* WesB (2.89)	75.9
H_bin_32 (3.51)	*Moritella dasanensis* ArB 0140 (4.89)	85.5
H_bin_39 (1.48)	*Sulfurimonas hongkongensis* (2.3)	85.6
I_bin_2 (1.85)	*Defluviitalea phaphyphila* (2.54)	75.6
Ice_3_bin_1 (1.27)	*Clostridium* sp Marseille P2434 (3.08)	80.2
Ice_3_bin_2 (3.71)	*Photobacterium phosphoreum* (4.55)	86.1
Ice_3_bin_3 (4.22)	*Photobacterium phosphoreum* (4.55)	97.6
Ice_3_bin_4 (3.24)	*Enterovibrio* sp. JCM 19048 (5.27)	77.7
Ice_5_bin_2 (3.74)	*Grimontia indica* (5.56)	77.5
Ice_7_bin_5 (4.47)	*Enterovibrio nigricans* DSM 22720 (5.04)	77.8
Ice_8_bin_6 (2.36)	*Brachyspira alvinipulli* ATCC 51933 (3.42)	76.1
Q_bin_5 (3.28)	*Enterovibrio norvegicus* FF 33 (5.16)	77.9
S3F1_bin_1 (2.42)	*Photobacterium phosphoreum* (4.55)	98.4
S3F2_bin_1 (1.92)	*Psychrilyobacter atlanticus* DSM 19335 (3.54)	76.5
S3F2_bin_4 (4.79)	*Enterovibrio norvegicus* FF 162 (5.05)	77.7
Scot_D_bin_18 (2.36)	*Arcobacter* sp L (2.95)	78.0
Scot_D_bin_7 (2.43)	*Brachyspira hyodysenteriae* (3.02)	76.3
Scot_D_bin_8 (2.32)	*Brachyspira alvinipulli* ATCC 51933 (3.42)	75.8
Scot_F_bin_12 (3.08)	*Grimontia indica* (5.56)	77.7
Scot_J_bin_10 (2.61)	*Odoribacter* sp. N54 MGS 14 (3.46)	76.5
Scot_J_bin_12 (3.04)	*Bacteroidales* bacterium Bact 02 (4.09)	77.5
W_bin_4 (2.15)	*Photobacterium phosphoreum* (4.55)	87.2
Water_bin_1 (2.15)	*Colwellia psychrerythraea* 34 H (5.37)	92.6
Water_bin_2 (2.15)	*Colwellia psychrerythraea* 34 H (5.37)	87.4
Z_bin_1 (3.43)	*Treponema* sp. CETP13 (2.54)	75.6

Drawing direct conclusions from comparisons of the typically smaller sized MAGs with larger complete reference genomes is difficult, however, the significant differences seen here between these deep-sea MAGs and their closest identified relatives gives an indication of possible adaptions of these microbes to life in the deep (**Supplemental Table 5**).^16^ Pressure can have an important influence on microbial life at a molecular level, and is found to influence DNA synthesis, protein structure and cell structure.^17–19^ Interestingly, when compared to their associated reference genomes, these MAGs were found to encode a greater relative abundance of genes involved in DNA replication, DNA repair, protein folding and cytoskeleton synthesis, all of which may represent adaptations of these microbes to the deep-sea (**Supplemental Table 5).**

The assembled deep-sea MAGs also encode a significantly greater relative abundance of genes associated with motility and chemotaxis (*p*= .0001) (**Supplemental Table 5**). Nutrient sources can be relatively sparse in the deep, where particulate organic matter (POM) descending from the surface can provide an important food source for microbes.^20^ In the open waters of the deep, motility may help to increase the likelihood of these bacteria encountering such POM.^21^ The importance of motility in the open ocean suggests that these MAGs encode genes that can help support life outside of the host, indicating a possible transient lifestyle rather than a reliance on host association.

### Bioluminescence

Bioluminescence can be an important strategy for deep-sea bacteria to establish themselves in the host microbiome, often forming symbiotic relationships in certain light organs of the host.^22^ Free-living microbes can also produce bioluminescence, these bacteria are often associated with POM and their bioluminescence indicates the presence of food to zooplankton and other fish. Once ingested, these bacteria can replicate in the more nutritious environment of the host gut and can often cause these smaller zooplankton to bioluminesce thus attracting predators. The host also provides a means for dissemination of the bacteria over a large area through their feces.^23^

The genes required for luciferase activity are encoded within the *lux* operon where the *luxA* and *luxB* genes encode for both subunits of the luciferase enzyme.^24,25^ Using a Hidden Markov model, it was possible to identify sequences related to the *luxA* luciferase genes in the metagenomic data. The distribution of these genes is much lower than anticipated, with only 12 of the fish samples and the water sample containing homologs to the *luxA* gene at a relatively low abundance (**Supplemental Table 6**). Many of the of identified *luxA* homologs (38.5%) were predicted to be encoded by members of the *Photobacterium* genus, specifically *Photobacterium phosphoreum¸* well-known for its bioluminescence, and *Photobacterium kishitanii –* a species closely related to the former which has previously been isolated from the light organ of deep-sea fishes.^26^ The relatively low level of luciferase-like genes across these samples was unexpected. If the theory that bioluminescence is used by these bacteria as an aid to become established in the gut of zooplankton and fish, then it could be expected that such luciferase-like clusters would be much more prevalent in the samples analyzed in this study.^27^ This strategy has been shown to be effective for zooplankton and smaller fish, however at higher trophic level where predators and prey are much larger, these results suggest that the bioluminescence produced by these microbes on POM and in the GI tracts of smaller zooplankton may not be sufficient to attract these larger fishes.

### Antibiotic resistance

The microbiome of animals can be an important source of antibiotic resistance genes that could potentially be horizontally transferred to pathogens.^28^ In addition, the marine environment has been implicated as a reservoir for antibiotic resistance.^29^ The deep-sea fish microbiome is an interesting dataset in this regard as the microbes here are distanced from human activity and thus may give an insight into the development of such resistance mechanisms in an environment where exogenous antibiotics are highly unlikely to occur and are even less likely to be present at inhibitory concentrations. It also gives an indication of competition between microbes in specific niches.

Bowtie2 was used to align the paired-end metagenomic reads from the deep-sea dataset against the MEGARes database in order to determine the abundance of known antibiotic resistance genes in the samples.^30^ Antibiotic resistance genes were found in less than half of all samples analyzed (Table 2). The resistance profile of these samples is dominated by resistance to a class of antibiotics known as elfamycins, which target elongation factor TU (EF-Tu) in bacterial cells.^31^ Potential resistance to elfamycins was identified in 20 of the metagenomic samples analyzed, in each case due to mutations in the EF-Tu encoding gene *tufA*. As most bacteria encode two virtually identical copies of the EF-Tu genes, levels of actual resistance due to differences in these genes may be overrepresented.^32^ As elfamycins are not used therapeutically, the mutations identified here may simply be a result of natural variation in the *tufA* genes rather than resistance due to the exposure to these antibiotics. EF-Tu has been identified as possibly having an important role in maintaining protein synthesis in response to high-pressure treatment of bacteria, thus potential adaptions of these genes to this environment may confer resistance to these antibiotics.^33^

**Table 2.** Type and abundance of antibiotic resistance genes identified in metagenomic samples expressed as raw hit counts and copies per million paired-end metagenomic reads (CPM)

Sample ID	Database	Class	Hits	CPM
A	Variant	Elfamycins	86	11.9
B	Variant	Elfamycins	2395	107.5
B	Variant	Rifampin	1263	56.7
C	NonVariant	Multi-drug Resistance	46	2.0
C	Variant	Elfamycins	159	6.8
C	Variant	Multi-drug Resistance	46	2.0
C2	Variant	Elfamycins	13	0.8
E	NonVariant	Betalactams	12	0.8
E	Variant	Betalactams	12	0.8
G	Variant	Elfamycins	2357	88.0
H	Variant	Elfamycins	3194	161.0
Ice_3	Variant	Elfamycins	1459	617.5
Ice_5	Variant	Elfamycins	145	49.8
Ice_7	NonVariant	Multi-drug Resistance	37	4.1
Ice_7	Variant	Elfamycins	2185	242.9
Ice_7	Variant	Multi-drug Resistance	37	4.1
Ice_8	Variant	Elfamycins	309	23.8
K	NonVariant	Multi-drug Resistance	292	17.1
K	Variant	Elfamycins	385	22.6
K	Variant	Multi-drug Resistance	292	17.1
S3F1	Variant	Elfamycins	137	38.6
S3F2	Variant	Elfamycins	329	88.0
Scot_D	Variant	Elfamycins	65	4.5
Scot_F	NonVariant	Multi-drug Resistance	22	2.3
Scot_F	Variant	Elfamycins	1795	184.3
Scot_F	Variant	Multi-drug Resistance	22	2.3
Scot_J	Variant	Elfamycins	1402	296.8
V	Variant	Elfamycins	295	14.8
W	Variant	Elfamycins	324	26.7
Water	Variant	Elfamycins	1239	481.8
Z	Variant	Elfamycins	490	24.5

When the screen for antibiotic resistance is restricted to genes associated with acquired resistance (non-variant) rather than resistance associated with SNPs in target genes (variant), the resistance profile of the samples is further diminished, with genes for acquired resistance only identified in five of the 47 intestinal samples. These results highlight the paucity of acquired antibiotic resistance in the microbiomes of these fishes.

The notably low incidence of detectable antibiotic resistance was verified using ABRicate (https://github.com/tseemann/abricate), to screen the assembled metagenomic contigs against the Comprehensive Antibiotic Resistance Database (CARD).^34^ This identified only two instances of AMR genes, both TEM-116 extended spectrum beta-lactamases, with >80% sequence identity and >75% coverage (**Supplemental Table 7**). No instances of antibiotic resistance were detected from the recovered MAGs when screened using this approach.

Antibiotic resistance in the marine environment is often associated with human intervention and pollution, which suggests that the isolated nature of the samples here would not favor the development of resistance.^35^ Whilst isolation from human activity is likely to play a role in the lack of resistance genes, other isolated environments have shown considerable levels of antibiotic resistance.^36^ It may also be, however, that the deep-sea fish microbiome possesses novel resistance mechanisms, which are yet to be identified in genetic screening.

## Conclusion

The life of deep-sea marine microbes is shaped by high pressure, darkness, low temperatures and low nutrient availability, all of which create a harsh environment for free-living cells. The intestines of deep-sea fish provide one of the few nutrient-dense environments in such habitats and, supported by our observations that the majority of the microbiome could not be assigned to lower taxonomic levels, the microbes that reside here are very different from surface microbes. The genes and pathways encoded by these microbial genomes must function in a low-temperature, high-pressure environment, and represent a novel cache of genetic information that could be utilized in biotechnology for use in biological processes and other applications conducted in harsh environments.

The MAGs assembled in this study offer a window to the lifestyles of these unique microbes, and comparison of their potential functionality shows a diversity of lifestyles. The adaptations of these deep-sea microbes are obvious when a comparison is made with the genomes of their most closely related surface microbes. A greater ability to deal with pressure-related stress is likely to be a result of their significantly increased capacity for DNA synthesis and protein folding. While the study is reliant on genomic comparisons, additional culture-based studies may be warranted on such strains to further understand how these potential genomic adaptions aid survival in this environment.

The levels of bioluminescent genes (*luxA*) were quite low, an unexpected finding given their potential importance in the life cycle of many marine microbes.^23^ This questions the extent to which free-living microbes may use bioluminescence to gain access to the intestine of these larger deep-sea fishes. Antibiotic resistance genes were not identified in the majority of the microbiomes, highlighting that the microbiomes of these fish represent a community of microbes assembled with little human interference.^37^

This study gives us a first glimpse of the compositional and functional diversity of microbes harbored in the intestines of deep-sea fish. While the diversity of deep-sea fish is difficult to capture as a whole, this study gives a snapshot of the microbes these may harbor across species and locations. We have identified a previously untapped potential source of novel compounds and processes which warrants further exploration and exploitation. Thus, whilst these deep-sea fish themselves often provokes much interest and enthusiasm; the bacteria they harbor within also appear to be as uniquely well adapted to life in the deep.

## Materials and methods

### Sample collection and DNA isolation

Fish were caught from the offshore waters of Ireland (September 2016), Scotland (59.4427 − 10.1123, September 2016), Iceland (October 2016) and in international waters off the Grand Banks of Newfoundland (May 2017) at a depth of approximately 1000 m, eight samples were caught at a depth of 850 m. Once caught, the intestinal contents from these fishes were isolated and stored for analysis using the OMNIgene GUT kit (DNA genotek, Ontario Canada). Total DNA was extracted from the stored samples using the PowerFecal DNA Isolation kit as per the manufacturer’s protocol (Qiagen). A water sample was also taken at a depth of >1000 m. 2 liters were filtered through a 0.45 µm pore size filter, and extraction of DNA from the filter material was carried out with the PowerFecal DNA Isolation kit (Qiagen).

### Whole-metagenome shotgun sequencing

The Nextera XT DNA Library Preparation kit (Illumina) was used for the preparation of whole-metagenome shotgun libraries. NextSeq libraries were sequenced on the Illumina NextSeq 500, with a NextSeq 500/550 High Output Reagent Kit v2 (300 cycles), in accordance with the standard Illumina sequencing protocols resulting in over 600 million read pairs.

### Sequencing and quality control

NextSeq libraries were sequenced on the Illumina NextSeq 500, with a NextSeq 500/550 High Output Reagent Kit v2 (300 cycles), in accordance with the standard Illumina sequencing protocols resulting in over 600 million read pairs. Raw reads were converted from FastQ to BAM format using Picard Tools (v. 2.7.1) and SAMtools (v. 1.5)^38^ and duplicate reads were removed using Picard Tools (https://github.com/broadinstitute/picard). Low-quality reads were removed using the trimBWAstyle.usingBam.pl script from the Bioinformatics Core at UC Davis Genome Center (https://github.com/genome/genome/blob/master/lib/perl/Genome/Site/TGI/Hmp/HmpSraProcess/trimBWAstyle.usingBam.pl). Specifically, bases with a quality score less than Q30 were trimmed and resulting reads shorter than 105bp were discarded.

Panphlan (v. 1.2.2.2)^39^ was used to build a pangenome database of all freely available fish genome sequences. Host contamination was identified by aligning the quality-trimmed whole-metagenome sequencing reads to this pangenome database with Bowtie2 (v. 2.2.9).^40^ The resulting alignments were converted to BAM format and host reads removed by SAMtools before conversion to FastQ format using BEDtools.^41^ FastQ files were converted to FastA using the fq2fa script packaged with IDBA-UD (v. 1.1.1).^42^

### Statistical analysis

Statistical analysis was performed in R (v. 3.4.4) (https://www.R-project.org/) and significance was accepted as *p* < .05 (FDR-adjusted). Figures were generated using the pheatmap (v. 1.0.10) (https://CRAN.R-project.org/package=pheatmap), ggplot2 (v. 2.2.1)^43^ and cowplot (v. 0.9.2) (https://CRAN.R-project.org/package=cowplot) packages for R.

### Composition

Taxonomic classification of paired-end reads was performed by comparison against the GTDB_r89_54k database developed by Méric and Wick^44^ using Kraken2 (v. 2.0.8beta)^10^ and relative abundances were sum-normalized at each taxonomic rank to account for inter-sample variation of sequencing depth. Functional analysis was performed using SUPER-FOCUS (v. 0.27)^45^ with DIAMOND alignment against the default DB_90 database.

### Reconstruction of metagenome-assembled genomes

Assembly of metagenomes was performed using the ‘meta-sensitive’ preset option of megahit (v. 1.1.2).^46^ Binning of metagenome-assembled genomes was performed using the ‘supersensitive’ preset option of MetaBat2 (v. 2.12.1).^47^ The completeness and contamination of these bins were assessed using the ‘lineage_wf’ workflow of CheckM (v. 1.0.7).^48^ MAGs with completeness ≥80% and contamination ≤5% were deemed ‘high-quality’. Initial phylogenetic analysis and taxonomic identification was performed using GTDB-tk.^49^ MAG length was calculated using the kmercountexact.sh script from BBTools (v.38.22) (https://sourceforge.net/projects/bbmap/).

To identify possible adaptations for survival in the gut of deep-sea fish, all high-quality MAGs were compared to the RefSeq and MarDB databases using FastANI. All MAGs and their closest reference genome, where ANI was greater than 75%, were annotated using Prokka (v. 1.12).^50^ The .ffn files generated for both MAGs and reference genomes were used as the input for functional analysis of ORFs using SUPER-FOCUS.

A paired t-test in R was used to detect statistically significant differences between the functional potential of these MAGs and their corresponding reference genomes. This was performed on SEED levels 1, 2, and 3 of the SUPER-FOCUS output.

The abundance of the 111 high-quality MAGs in the metagenomes was determined by aligning all paired-end reads against these MAGs using Bowtie2 using thevery-sensitive preset. The percentage of the paired-end reads which mapped to each MAG was determined using the idxstats script in SAMtools.

Percentage of Conserved Proteins (PoCP) was calculated as described by Qin *et al*.^14^ Network analysis was performed in R using the igraph package (v.1.2.6).^51^

### Antimicrobial resistance

Read-level antimicrobial resistome analysis was performed by Bowtie2 alignment against the MEGARes database.^30^ The resulting SAM files were summarized at gene, group, class, and mechanism level using the Resistome Analyzer pipeline (https://github.com/cdeanj/resistomeanalyzer). Reads were normalized for sequencing depth across samples as copies per million reads (CPM). Additional contig-level analysis was performed using ABRicate screening using the packaged CARD database. Abricate homology was accepted as reported hits with >75% coverage and >80% identity.

### Bioluminescence

Assembled metagenomes were translated in all six reading frames using the EMBOSS (v. 6.6.0) transeq script with option -frame 6 and were searched using Hidden Markov Models obtained from the pfam database.^52^ PF00296 was used to search for luciferase activity. The Hmmer (v. 3.1) (http://hmmer.janelia.org/) hmmsearch script was used to identify potential matches using an e-value cutoff of 0.1. The abundance of each contig containing a significant hit was calculated by aligning all reads against the assembled metagenomes using Bowtie2 and calculating coverage using the SAMtools idxstats script. These contigs were taxonomically classified using Kraken2 and the same GTDB database used for taxonomic profiling of paired-end reads.

## Supplementary Material

Supplemental Material
