# Supplementary material for: The microbiome of deep-sea fish reveals new microbial species and a sparsity of antibiotic resistance genes
Source: Gut Microbes. 2021 May 10;13(1):1921924. doi: 10.1080/19490976.2021.1921924 (PMC8115496; doi:10.1080/19490976.2021.1921924)
Supplement: Supplemental Material [file KGMI_A_1921924_SM9825.zip › Supplementary information/Supplementary Materials.docx]

**Supplementary Materials for**

The Microbiome of Deep-Sea Fish Reveals New Microbial Species and a Sparsity of Antibiotic Resistance Genes

Fergus W. J. Collins, Calum J. Walsh, Beatriz Gomez-Sala, Elena Guijarro-García, David Stokes, Klara B. Jakobsdóttir, Kristján Kristjánsson, Finlay Burns, Paul D. Cotter, Mary C. Rea, Colin Hill and R. Paul Ross

Correspondence to: [p.ross@ucc.ie](mailto:p.ross@ucc.ie)

**Supplemental Figure 1.** PCoA plot displaying diversity in SUPER-FOCUS level 1 results between MAGs and reference genomes. Those MAGs that have closely related reference genomes are connected by lines.

| **Fish Species** | **Sample ID** |
| --- | --- |
| *Polycanthonotus rissoanus* | A |
| *Bathypterois dubius* | B |
| *Notacanthus chemnitzii* | C |
| *Anoplogaster cornuta* | E |
| *Polycanthonotus rissoanus* | F |
| *Coryphaenoides rupestris* | G |
| *Gaidropsaurus ensis* | H |
| *Centroscymnus coelolepis* | I |
| *Anoplogaster cornuta* | J |
| *Notacanthus chemntzii* | K |
| *Polycanthonotus rissoanus* | M |
| *Bathysaurus ferox* | N |
| *Simenchelys parasitica* | O |
| *Centroscyllium fabricii* | P |
| *Alepocephalus agassizii* | Q |
| *Eurypharynx pelecanoides* | R |
| *Apristurus sp.* | S |
| *Stomias boa boa* | U |
| *Alepocephalus bairdii* | V |
| *Bathysaurus ferox* | W |
| *Bathylagus euryops* | X |
| *Centroscymynus coelolepis* | Y |
| *Scopelosaurus lepidus* | Z |
| *Hydrolagus affinis* | A2 |
| *Pachystomias microdon* | B2 |
| *Cottunculus thomsonii* | C2 |
| *Lampadena speculigera* | D2 |
| *Pachystomias microdon* | E2 |
| *Mora moro* | Scot_A |
| *Argentina silus* | Scot_B |
| *Aphanopus carbo* | Scot_C |
| *Alepocephalus bairdii* | Scot_D |
| *Galeus murinus* | Scot_E |
| *Coryphaenoides rupestris* | Scot_F |
| *Chimaera monstrosa* | Scot_I |
| *Halargyreus johnsonii* | Scot_J |
| *Macrourus berglax* | Ice_2 |
| *Lepidion eques* | Ice_3 |
| *Trachyrincus murrayi* | Ice_5 |
| *Trachyrincus murrayi* | Ice_7 |
| *Alepocephalus bairdii* | Ice_8 |
| *Molva dypterygia* | S5F1 |
| *Alepocephalus bairdii* | S2F2 |
| *Coelorinchus occa* | SSGF1 |
| *Lepidion eques* | S3F2 |
| *Alepocephalus bairdii* | BS1 |
| *Lepidion eques* | S3F1 |

**Supplemental Table 1.** List of fish species sampled for microbiome analysis and corresponding sample ID.

| **Sample ID** | **Read Pairs Mapped to MAGs (%)** |
| --- | --- |
| A | 7.09% |
| A2 | 0.07% |
| B | 10.13% |
| B2 | 0.07% |
| BS1 | 6.08% |
| C | 4.88% |
| C2 | 0.23% |
| D2 | 0.05% |
| E | 0.02% |
| E2 | 0.10% |
| F | 0.35% |
| G | 24.79% |
| H | 44.05% |
| I | 1.40% |
| Ice_2 | 0.07% |
| Ice_3 | 62.23% |
| Ice_5 | 19.46% |
| Ice_7 | 58.42% |
| Ice_8 | 13.31% |
| J | 0.01% |
| K | 78.01% |
| M | 0.13% |
| N | 0.03% |
| O | 0.01% |
| P | 0.05% |
| Q | 1.30% |
| R | 0.03% |
| S | 0.47% |
| S2F2 | 3.22% |
| S3F1 | 4.74% |
| S3F2 | 14.42% |
| S5F1 | 0.02% |
| Scot_A | 0.06% |
| Scot_B | 0.01% |
| Scot_C | 0.01% |
| Scot_D | 17.76% |
| Scot_E | 0.16% |
| Scot_F | 35.96% |
| Scot_I | 0.01% |
| Scot_J | 53.67% |
| SSGF1 | 0.02% |
| U | 0.01% |
| V | 4.08% |
| W | 2.25% |
| X | 0.02% |
| Y | 0.14% |
| Z | 38.59% |

**Supplemental Table 4.** Proportion of microbiome comprised of the total MAGs assembled in this study

| **Genus (Hits per Mbp of Assembled Metagenome)** | | | | | | | | | | |
| --- | --- | --- | --- | --- | --- | --- | --- | --- | --- | --- |
| **Sample** | ***Aliivibrio*** | ***Enterovibrio*** | ***Photobacterium*** | ****Unclassified** | ***Shewanella*** | ***Peptoniphilus*_A** | ***Not Classified** | ***56-14-T64*** | ***Colwellia*** | ***Poseidonibacter*** |
| B | 0.001836483 | 0.001836483 | 0.001836483 | 0 | 0 | 0 | 0 | 0 | 0 | 0 |
| BS1 | 0 | 0 | 0 | 0.008053724 | 0 | 0 | 0 | 0 | 0 | 0 |
| C | 0 | 0 | 0 | 0.001334867 | 0 | 0 | 0 | 0 | 0 | 0 |
| Ice_3 | 0 | 0 | 0.033906496 | 0 | 0 | 0 | 0 | 0 | 0 | 0 |
| Ice_7 | 0.007493966 | 0 | 0 | 0 | 0.007493966 | 0 | 0 | 0 | 0 | 0 |
| S3F1 | 0 | 0 | 0.016715782 | 0 | 0 | 0 | 0 | 0 | 0 | 0 |
| S3F2 | 0.021972406 | 0 | 0 | 0 | 0 | 0 | 0 | 0 | 0 | 0 |
| Scot_D | 0 | 0 | 0 | 0 | 0 | 0.00286778 | 0 | 0 | 0 | 0 |
| Scot_F | 0 | 0 | 0 | 0 | 0 | 0 | 0.00455002 | 0 | 0 | 0 |
| Scot_J | 0.010845294 | 0 | 0.032535883 | 0 | 0 | 0 | 0 | 0 | 0 | 0 |
| W | 0 | 0 | 0.006001392 | 0 | 0 | 0 | 0 | 0 | 0 | 0 |
| Water | 0 | 0 | 0 | 0.035532025 | 0 | 0 | 0 | 0.035532025 | 0.035532025 | 0.071064051 |
| Z | 0 | 0 | 0.003143252 | 0 | 0 | 0 | 0 | 0 | 0 | 0 |

*Not Classified = could not be classified to a genus level but could be classified to a higher phylogenetic category. **Unclassified = unable to classify to any phylogenetic category

**Supplemental Table 6.** Relative abundance and phylogenetic breakdown of homologs of the *luxA* gene identified by HMM screening.
